# IgG4-associated hypophysitis coexisting with MALT lymphoma and gangliocytoma: first case report and literature review

**DOI:** 10.3389/fneur.2023.1253821

**Published:** 2023-11-09

**Authors:** Dongbo Zou, Li Jiang, Tao Yang, Jingmin Cheng, Yuan Ma

**Affiliations:** ^1^Department of Neurosurgery, General Hospital of Western Theater Command, Chengdu, China; ^2^Department of Rheumatology and Immunology, The Second People's Hospital of Chengdu, Chengdu, China

**Keywords:** IgG4-related hypophysitis, MALT lymphoma, gangliocytomas, sellar region, headaches, vision loss

## Abstract

IgG4-related or IgG4-associated hypophysitis is a rare disease characterized by the infiltration of IgG4-positive plasma cells into pituitary gland tissue. Gangliocytomas in the sellar region are also extremely rare and are associated with pituitary adenomas in the majority of cases. Sellar mucosa-associated lymphoid tissue (MALT) lymphoma is an exceedingly rare condition. In this study, we present a case of IgG4-associated hypophysitis coexisting with MALT lymphoma and gangliocytoma. However, to elucidate the potential pathophysiological relationship, it is imperative to gather additional cases of IgG4-related hypophysitis accompanied by MALT lymphoma and gangliocytoma.

## Introduction

IgG4-related hypophysitis, a rare condition affecting the sellar region, has been increasingly reported in recent years. It can manifest as an isolated disease or as part of a multisystemic disorder. However, it is worth noting that the majority of IgG4-related hypophysitis cases are not associated with other neoplastic diseases (1).

In this report, we present a biopsy-confirmed case of IgG4-associated hypophysitis coexisting with MALT lymphoma and gangliocytoma.

## Case

**Figure 1:**
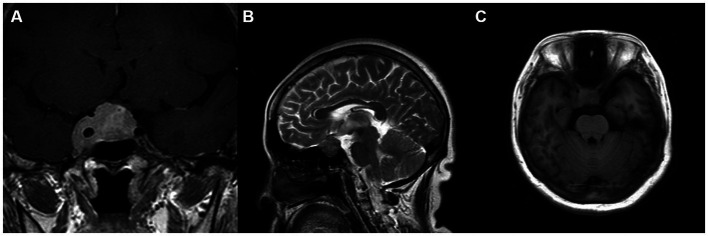
**(A–C)** The first MRI examination showed that the lesion invaded the cavernous sinus, with obvious enhancement after enhancement and compression of the optic chiasm. **(A)** Coronal T1 enhancement; **(B)** Sagittal T2 imaging; and **(C)** Axial T1 imaging.

In May 2018, a 53-year-old female presented with a 1-month history of worsening right visual acuity and headaches. Prior to this, she had been in good health. Endocrine assessment revealed abnormal secretion of prolactin and thyroid hormone (Table 1). MRI examination of the pituitary gland revealed a well-defined soft tissue mass measuring 3.2 cm × 1.9 cm × 1.9 cm, located in sella, suprasellar area, and right cavernous sinus. Additionally, there were small patchy short T1 signal shadows within the lesion, which displayed heterogeneous enhancement. The mass encased the right internal carotid artery and compressed the right optic chiasm, resulting in an unclear pituitary stalk (Figure 1). Visual examination showed decreased vision and visual field defects in the right eye, whereas the left eye exhibited normal vision. Surgical resection was recommended to alleviate optic nerve compression. However, the patient declined surgery and opted for continued follow-up.

**Table 1:** Endocrine assessment for the first time.

Report projects	Results	Normal values	Unit
Prolactin	86.71	3.34–26.7	ng/mL
FT3	2.33	2.38–4.34	pg/mL
FT4	0.68	0.9–1.76	ng/dL
Cortisol	7.0 (08:00)−2.37 (16:00)−4.61 (00:00)	Morning 6.7–22.6 μg/dL; Afternoon 0–10 μg/dL	Ug/dL

**Figure 2:**
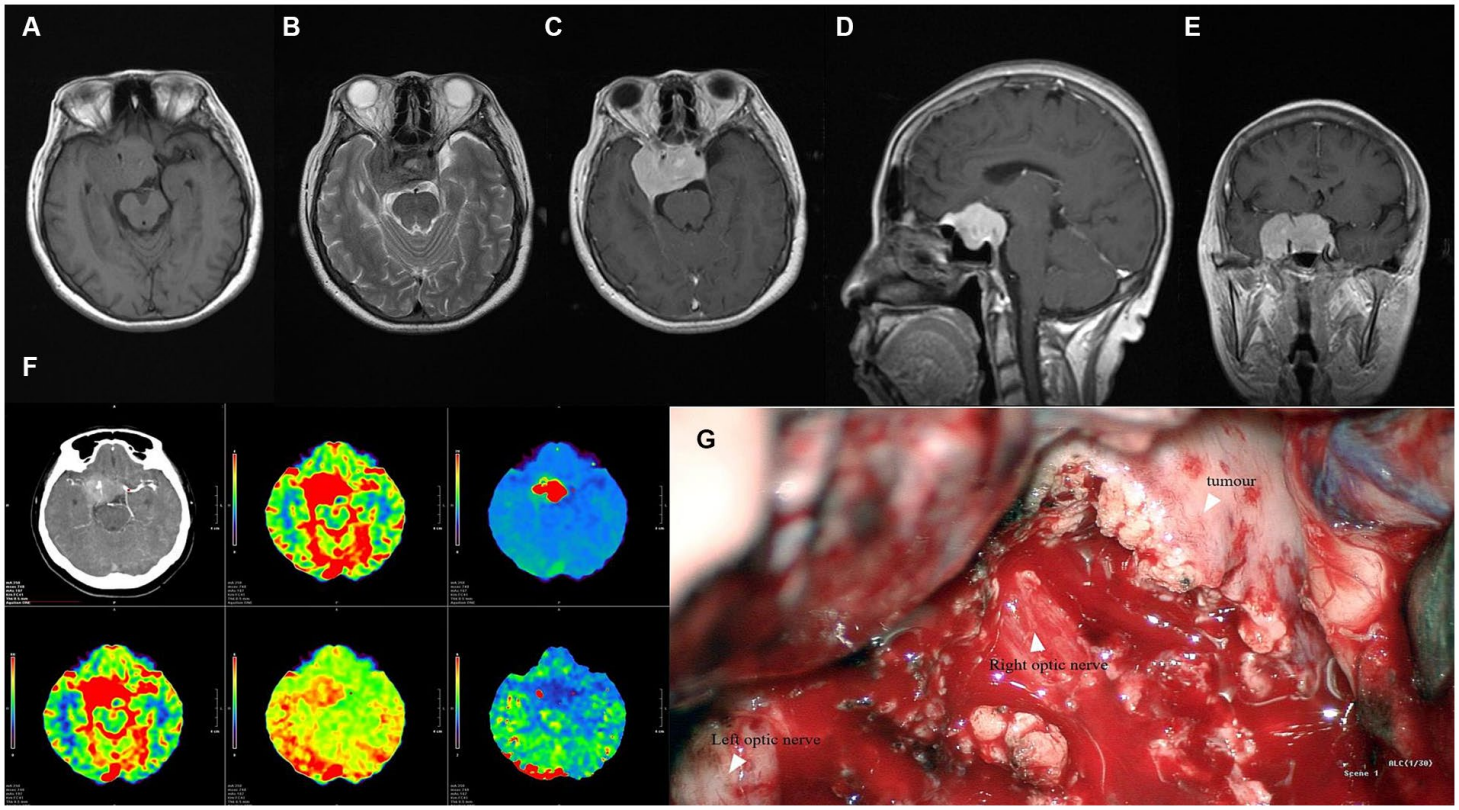
**(A,B)** MRI plain scan showed suprasellar irregular soft tissue signal mass; **(C–E)** Enhanced MRI scan showed that the lesion was significantly enhanced, the adjacent meninges were enhanced, and the boundary was clear. The mass wrapped the cavernous sinus and the cavernous sinus segment of the internal carotid artery on both sides, broke through the sellar septum upward, and pushed the optic chiasm; **(F)** CTA showed abundant blood perfusion in the lesion; **(G)** During the operation, as we can see, the boundary of the lesion was clear, the bilateral internal carotid artery was wrapped, and the blood supply was abnormally rich.

In January 2019, the patient was readmitted due to severe binocular vision loss and debilitating headaches. Ophthalmic vision examination revealed further deterioration in the right eye, with vision reduced to perceiving only light. Simultaneously, vision in the left eye was limited to half a meter in front. Pituitary hormone assessment indicated persistently disturbed prolactin and thyroid hormone secretion (Table 2). MRI examination demonstrated significant enlargement of the sellar and suprasellar lesions, now measuring 4.7 cm × 2.8 cm × 4.8 cm, with uniform enhancement following contrast administration. Adjacent meningeal enhancement was also observed. The mass encased the cavernous sinuses and internal carotid arteries bilaterally, extending superiorly through the sellar septum and compressing the optic chiasm (Figure 2). Physical examination, Chest CT, and abdominal ultrasound did not found any mass or other abnormal.

**Table 2:** Endocrine assessment before surgery: pituitary function deteriorated further.

Report projects	Results	Normal values	Unit
Prolactin	0.11	3.34–26.7	ng/mL
FT3	1.97	2.38–4.34	pg/mL
FT4	0.70	0.9–1.76	ng/dL
Cortisol	7.54 (08:00)−4.87 (16:00)−3.61 (00:00)	Morning 6.7–22.6 μg/dL; Afternoon 0–10 μg/dL	μg/dL

Given the rapid decline in vision and severe headaches of the patient. A craniotomy procedure via pterional approach was performed for the “tumor” resection. During the procedure, we observed that the lesion exhibited rich blood supply, firm texture, and complete encasement of the optic nerve and internal carotid artery. Consequently, a subtotal resection was performed. Postoperatively, the patient received a physiologic dose of hydrocortisone. Although she experienced complications such as diabetes insipidus and hypernatremia in the perioperative period, pituitary hormone assessment gradually returned to normal. Furthermore, postoperative headaches were alleviated, and partial restoration of vision was achieved.

**Figure 3:**
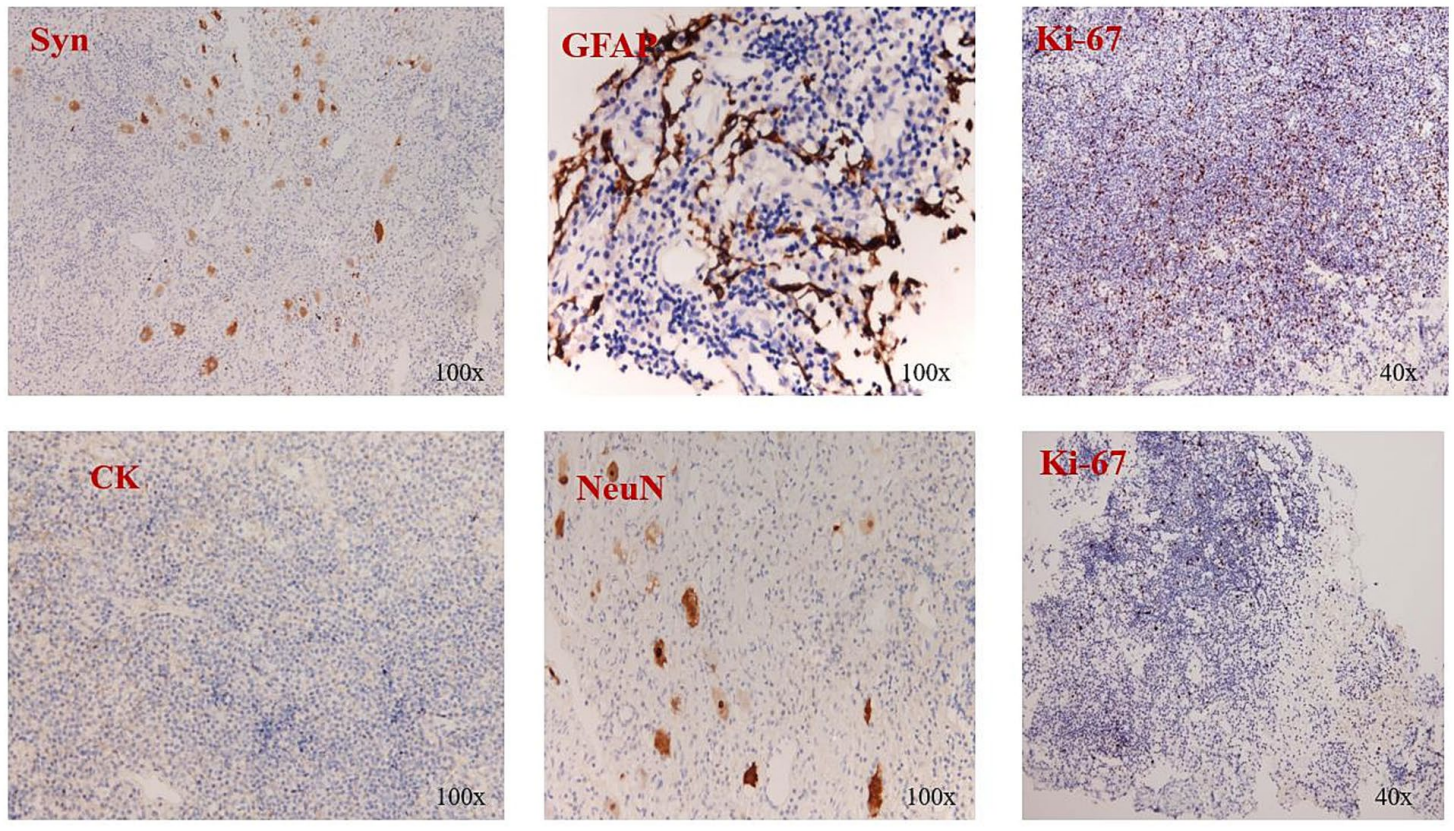
Immunohistochemical staining showed the Syn (−), CK (−), GFAP (−), NeuN (−), and Ki-67 (+ 20%).

**Figure 4:**
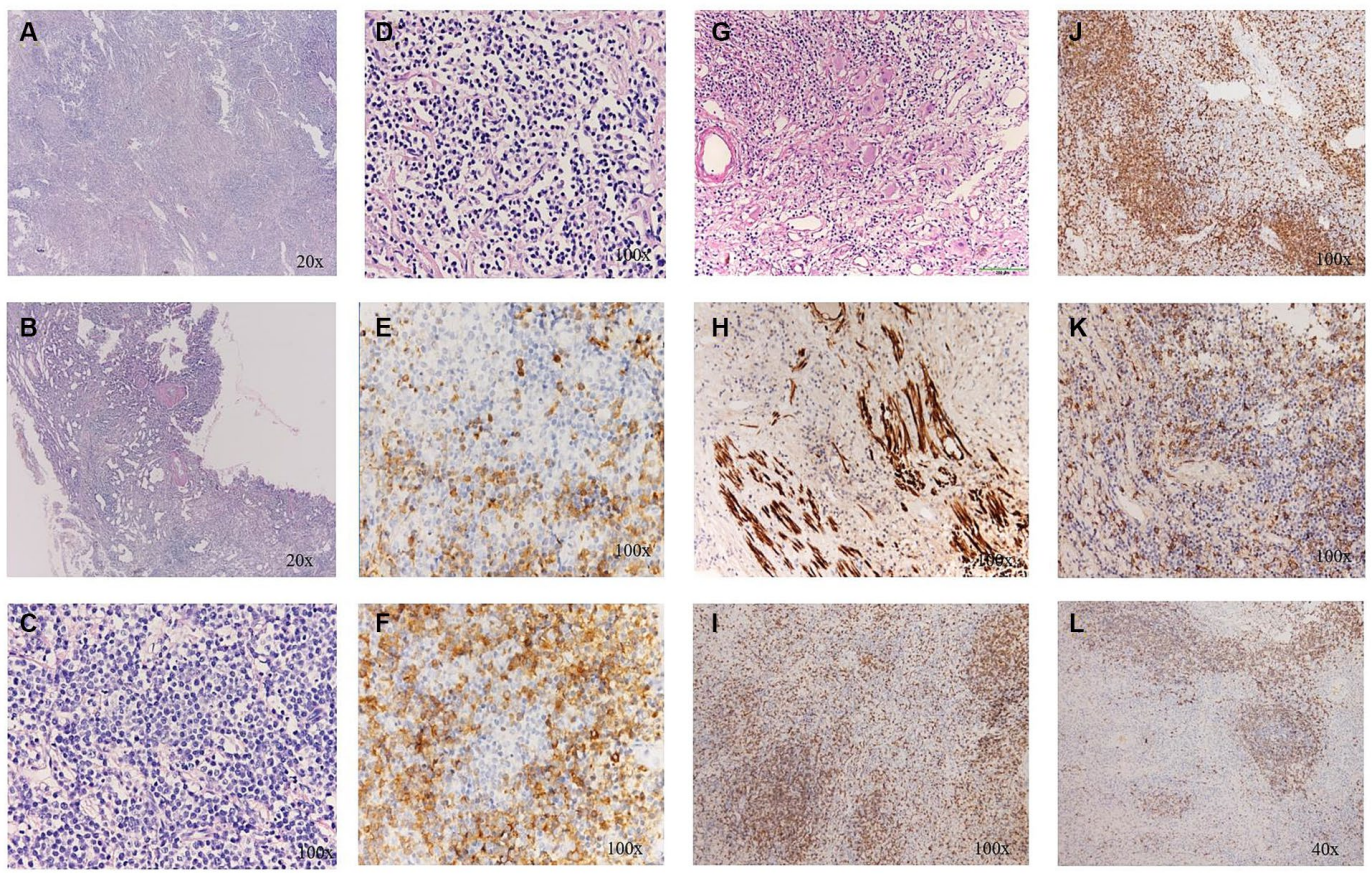
**(A)** The distinct fibrosclerotic background; **(B)** Sclerosis and hyalinization of the wall of small vessels; **(C)** A large number of lymphocytes; **(D)** Patches of plasma cells; **(E,F)** More than 50 IgG4positive cells per high-power field. **(G)** Ganglion cells under H&E staining; **(H)** S-100 positivity; **(I)** CD 3(+); **(J)** CD 4(+); **(K)** CD 20(+); and **(L)** CD 21(+).

On pathological examination, we initially performed immunohistochemical staining for CD56, syn, and cga to confirm the presence of neuroendocrine tumors; however, the results were negative. Subsequently, we continued to test for CK8/18 and CK to verify the presence of meningioma, and the results remained negative. Next, we performed immunohistochemical staining for GFAP to confirm the presence of glioma, and the results were again negative (Figure 3). After ruling out common lesions in the sella region, we suspected that the lesion was inflammatory. The next pathological examination revealed that the lesion was predominantly composed of lymphoid cells and plasma cells. Notably, there were more than 50 IgG4-positive plasma cells per high-power field (HPF), accounting for over 50% of the IgG-positive plasma cells. Additionally, numerous ganglion cells were identified in the specimens. These ganglion cells displayed scattered distribution, large nuclei, and abundant basophilic cytoplasm. The HE staining revealed the presence of ganglia, while immunohistochemical staining demonstrated positive labeling for S-100. Furthermore, immunohistochemistry analysis revealed positive expression of CD3, CD4, CD20, CD21 K(+), and λ(+) (Figure 4). Based on these immunohistochemical findings, including plasma cell positivity, the presence of CD3 and CD4 in the background, and an enlarged and distorted follicular network demonstrated by CD21, we were also able to confirm a diagnosis of MALT lymphom. Based on these findings, the final diagnosis was IgG4-associated hypophysitis coexisting with MALT lymphoma and gangliocytoma. The serum immunoglobulins level was not evaluated during the whole course.

Following the definitive diagnosis, the patient was initiated on high-dose glucocorticoid therapy: prednisone 40 mg/day for 4 weeks, followed by a tapering dose over the next 3 months. We have been monitoring the patient’s progress; unfortunately, she declined further serologic and imaging evaluations.

## Discussion

We present a unique case of biopsy-proven IgG4-associated hypophysitis coexisting with MALT lymphoma and gangliocytoma, which, to the best of our knowledge, has not been reported previously. Over a span of 6 months, the lesion in the sellar region rapidly enlarged, resulting in severe vision loss in both eyes. To preserve vision, the patient underwent emergency surgery, leading to substantial removal of the lesion. Pathological examination confirmed the diagnosis of IgG4-associated hypophysitis coexisting with MALT lymphoma and gangliocytoma. Subsequently, the patient received low-dose glucocorticoid treatment for a duration of 2 years. During a 3-year telephone follow-up, the patient reported significant improvement in vision. Unfortunately, the patient declined further imaging studies, preventing a comparison with preoperative images.

IgG4-related disease is a chronic, progressive inflammatory disorder characterized by fibrosis that can affect multiple organs. It can often be misdiagnosed as malignancy, infection, or other immune-mediated diseases. The disease is characterized by mass lesions, elevated serum IgG4 levels, infiltration of IgG4-positive plasma cells, and tissue fibrosis (2). At present, its pathogenesis is still unclear. Although IgG4-related disease rarely affects the brain parenchyma, the pituitary gland is one of its targets. IgG4-related hypophysitis typically presents with symptoms such as headache, visual field defects, and vision loss, which are caused by mass effect in the sellar region. Impaired pituitary function can lead to adenohypophysis and central diabetes insipidus. It is important to differentiate IgG4-related hypophysitis from other conditions such as Langerhans cell histiocytosis (LCH), sarcoidosis, granulomatosis with polyangiitis, infections, and tumors (3), so pathological diagnosis is the gold standard to distinguish them. In view of the scarce prevalence of IgG4-related hypophysitis, various countries and regions utilize their respective diagnostic samples, leading to a lack of standardized guidelines. In this particular scenario, we have relied upon Leporati’s diagnostic criteria for reference (Table 3) (4).

**Table 3:** Diagnostic criteria for IgG4-related hypophysitis.

Diagnostic criteria for IgG4-related hypophysitis
Criterion 1: Pituitary histopathology
Mononuclear infiltration of the pituitary gland, rich in lymphocytes and plasma cells, with more than 10 IgG4- positive cells per high-power field
Criterion 2: Pituitary MRI
Sellar mass and/or thickened pituitary stalk
Criterion 3: Biopsy-proven involvement in other organs
Association with IgG4-positive lesions in other organs
Criterion 4: Serology
Increased serum IgG4 (140 mg/dL)
Criterion 5: Response to glucocorticoids
Shrinkage of the pituitary mass and symptom improvement with steroids.
Diagnosis of IgG4-related hypophysitis is established when any of the following is fulfilled:
Criterion 1
Criteria 2 and 3
Criteria 2, 4, and 5

For the diagnosis of the case, firstly, in the histopathological examination, the presence of mononuclear infiltration in the pituitary gland, characterized by abundant lymphocytes and plasma cells, with over 10 IgG4-positive cells per high-power field, fulfills Leporati’s diagnostic criterion 1. Secondly, the patient underwent MRI examination, revealing the presence of a mass in the sellar region. Although no thickening of the pituitary stalk was identified, this finding largely satisfies Leporati’s diagnostic criterion 2. Thirdly, our patient received glucocorticoid therapy, and although no imaging examination was conducted, there was evident improvement in symptoms, which also substantially meets Leporati’s diagnostic criterion 5. Lastly, confirmation of the diagnosis requires meeting diagnostic criterion 1 alone, thus establishing this as a case of IgG4-related hypophysitis.

Differentiation of IgG4-related hypophysitis from other rare diseases in the sellar region is still necessary. One such disease is mixed pituitary adenoma-gangliocytoma, which predominantly affects female patients and is characterized by the secretion of one or more pituitary hormones, including growth hormone (GH) and prolactin (PLR), with a higher prevalence of growth hormone secretion (5). Diagnosis of this disease primarily relies on pathological examination. However, in our case, the patient did not display hormone hypersecretion, and the pathological examination did not reveal pituitary adenoma-associated cells, leading us to exclude the possibility of a mixed pituitary adenoma-gangliocytoma. Another infrequent sellar region disease is hypothalamic hamartoma, which is more commonly seen in infants and children. It is characterized by congenital abnormal brain tissue development and presents mainly with precocious puberty and epilepsy as clinical manifestations (6). Pathological examination of this disease shows an absence of solid tumor components but mainly consists of mature neurons and glial cells. Taking into account the patient’s age, medical history, and pathological findings, we ruled out this condition.

Glucocorticoids are currently recommended as the first-line treatment for IgG4-related disease, unless contraindications are present (7). Our patient responded well to glucocorticoid therapy, with significant improvement in pituitary function and partial recovery of vision. The use of glucocorticoids may vary in different regions. In most cases, prednisone at a dose of 30–40 mg/day is commonly used as an initial treatment, which is then gradually tapered over a period of 2–4 weeks. Glucocorticoid therapy is typically discontinued after 3–6 months. However, some researchers have suggested low-dose maintenance therapy with glucocorticoids for up to 3 years to maximize patient benefits (8).

Mucosa-associated lymphoid tissue (MALT) lymphoma, first reported in 1984, demonstrates a slow clinical progression (9). While MALT lymphoma primarily occurs in the stomach, recent studies have reported an increasing number of cases in ocular adnexal, cutaneous, pulmonary, and sella regions (10, 11). Sellar MALT lymphoma is an extremely rare condition. It is categorized as a low-grade malignant lymphoma distinguished by its unique etiology, pathological characteristics, and favorable prognosis. The pathogenesis of MALT lymphoma in the sellar region remains unknown, although several studies have suggested a link between MALT lymphoma and chronic infections and autoimmune diseases (12). Additionally, research has proposed a potential association between MALT lymphoma and IgG-related diseases (13). In this particular case, the presence of IgG4-related inflammation, plasma cells on pathological examination, and the expression of CD3 and CD4 cells in the background, as well as an enlarged and distorted follicular network on CD21 positivity, ultimately led to the diagnosis of MALT lymphoma.

Gangliocytomas are rare and benign neuronal cell tumors that are predominantly found in the hypothalamic and sellar regions. They often coexist with pituitary adenomas, mainly of the growth hormone (GH) type (14, 15). Since its first report by Kiyono in 1926, over 100 cases of pituitary adenoma combined with gangliocytoma have been reported worldwide (16–18). Currently, diagnosing gangliocytoma based on clinical and radiological elements is challenging. Moreover, due to its frequent association with pituitary adenomas, gangliocytoma is often overlooked in pathological examinations (19).

The origin of gangliocytoma remains unclear, and there are three hypotheses regarding its mechanism of development: (1) it originates from the hypothalamus and produces growth hormone-releasing hormone, leading to hyperplasia of pituitary cells (20, 21); (2) it originates from primitive stem/progenitor cells of the adenohypophysis (17, 22); (3) it arises from neuronal transformation within adenoma cells, as evidenced by intermediate structures observed between adenoma cells and ganglion cells in ultrastructural studies (23). However, the presence of IgG4-associated inflammation with ganglion cells and the absence of pituitary adenoma cells in our case do not align with any of these hypotheses. Therefore, we speculate that the cellular origin of ganglion cells may also be related to inflammatory or immune functions, which requires further investigation.

In conclusion, we report the first case of biopsy-proven IgG4-related hypophysitis accompanied by MALT lymphoma and gangliocytoma. Although the patient did not fully recover pituitary function and vision, her headaches were significantly relieved, and disease progression was prevented through surgical and medical interventions. We hope that our treatment approach can provide valuable insights and guidance for future cases.

## Data availability statement

The original contributions presented in the study are included in the article/supplementary material, further inquiries can be directed to the corresponding author.

## Ethics statement

The studies involving human participants were reviewed and approved by Ethics Committee of General Hospital of Western Theater Command. The patients/participants provided their written informed consent to participate in this study. Written informed consent was obtained from the patient for the publication of any potentially identifiable images or data included in this article.

## Author contributions

DZ: Investigation, Writing – original draft. LJ: Conceptualization, Writing – original draft. TY: Writing – review & editing. JC: Supervision, Validation, Writing – review & editing. YM: Project administration, Resources, Writing – review & editing.
